# Do work-related factors contribute to differences in doctor-certified sick leave? A prospective study comparing women in health and social occupations with women in the general working population

**DOI:** 10.1186/s12889-016-2908-1

**Published:** 2016-03-08

**Authors:** Cecilie Aagestad, Reidar Tyssen, Tom Sterud

**Affiliations:** Department of Occupational Health Surveillance, National Institute of Occupational Health, PO Box 8149 Dep, N-0033 Oslo, Norway; Department of Behavioral Sciences, Institute of Basic Medical Sciences, Faculty of Medicine, University of Oslo, PO Box 1111, Blindern, 0317 Oslo, Norway

**Keywords:** Absenteeism, Female worker, Health and social sector, Health and social worker, Mechanical factor, Psychosocial factor, Risk factor, Sickness absence

## Abstract

**Background:**

Doctor –certified sick leave is prevalent in the health and social sector. We examined whether the higher risk of doctor-certified sick leave in women in health and social occupations compared to women in other occupations was explained by particular work-related psychosocial and mechanical risk factors.

**Methods:**

A randomly drawn cohort aged 18–69 years from the general population in Norway was surveyed in 2009 (*n* = 12,255, response at baseline = 60.9 %), and was followed up in the national registry of social transfer payments in 2010. Eligible respondents were women registered with an active employee relationship for ≥100 actual working days in 2009 and 2010 (*n* = 3032). Using this sample, we compared health and social workers (*n* = 661) with the general working population (*n* = 2371). The outcome of interest was long-term sick leave (LTSL) ≥21 working days during 2010. Eight psychosocial and eight mechanical factors were evaluated.

**Results:**

After adjusting for age, previous LTSL, education and working hours/week, women in health and social occupations had a higher risk for LTSL compared with women in the general working population (OR = 1.42, 95 % CI = 1.13–1.79; *p* = 0.003). After adjusting for psychosocial and mechanical factors, 70 % of the excess risk for LTSL was explained compared with the initial model. The main contributory factors to the increased risk were threats of violence and violence, emotional demands and awkward lifting.

**Conclusions:**

Psychosocial and mechanical factors explained much of the excess risk for LTSL in women in health and social occupations compared with working women in general. Psychosocial risk factors were the most important contributors.

## Background

Doctor –certified sick leave is prevalent in the health and social sector [1, 2]. According to national statistics women in health and social occupations have a higher risk of sick leave compared with women in the general Norwegian working population [2], but the explanation for this increased risk is poorly understood. Thus, identifying specific work environment factors that might explain this greater risk of long-term sick leave (LTSL) will be important for developing interventions that aim to reduce sick leave in this sector.

In the health and social sector, the work environment includes specific psychosocial and mechanical factors related to patient handling activities, such as emotional demands [3], violence and threats of violence [4, 5], and lifting patients [6]. Prospective studies of health and social workers have identified several psychosocial risk factors for LTSL [4, 7, 8]. A Danish study of health workers in eldercare services found that emotional demands, role conflict and low job control were the most important predictors of LTSL [7]. In an earlier study of the health and social workers cohort investigated in the present study, we found that violence and threats of violence was the strongest predictor for LTSL among female health and social workers in Norway [9]. We also identified one prospective study from 2005 that attempted to explain the difference in sick leave between occupational groups according to work environment factors, where 52 % of the variation in sickness absence days between workplaces in municipal care, technical services and a pharmaceutical company was explained by psychosocial factors [10].

Mechanical risk factors related to patient care such as heavy lifting and lifting in awkward body postures have also been identified as predictors of LTSL [6, 11, 12]. A small number of prospective studies have reported that those who work in occupations that involve handling patients or clients have a higher risk of LTSL compared with other occupational groups [1, 13]. However, previous studies have not investigated the extent to which psychosocial and mechanical work environmental factors might contribute to the greater risk of LTSL for women in health and social occupations compared with women in other occupations, and whether psychosocial or mechanical factors are considered the most important.

Thus, in the present study, we investigated whether the higher risk for LTSL (doctor-certified sick leave ≥21 days) among women in health and social occupations compared with women in the general working population could be explained by considering several mechanical risk factors and psychosocial risk factors, which have been reported previously as predictors of LTSL in this sector.

## Methods

**Fig. 1:**
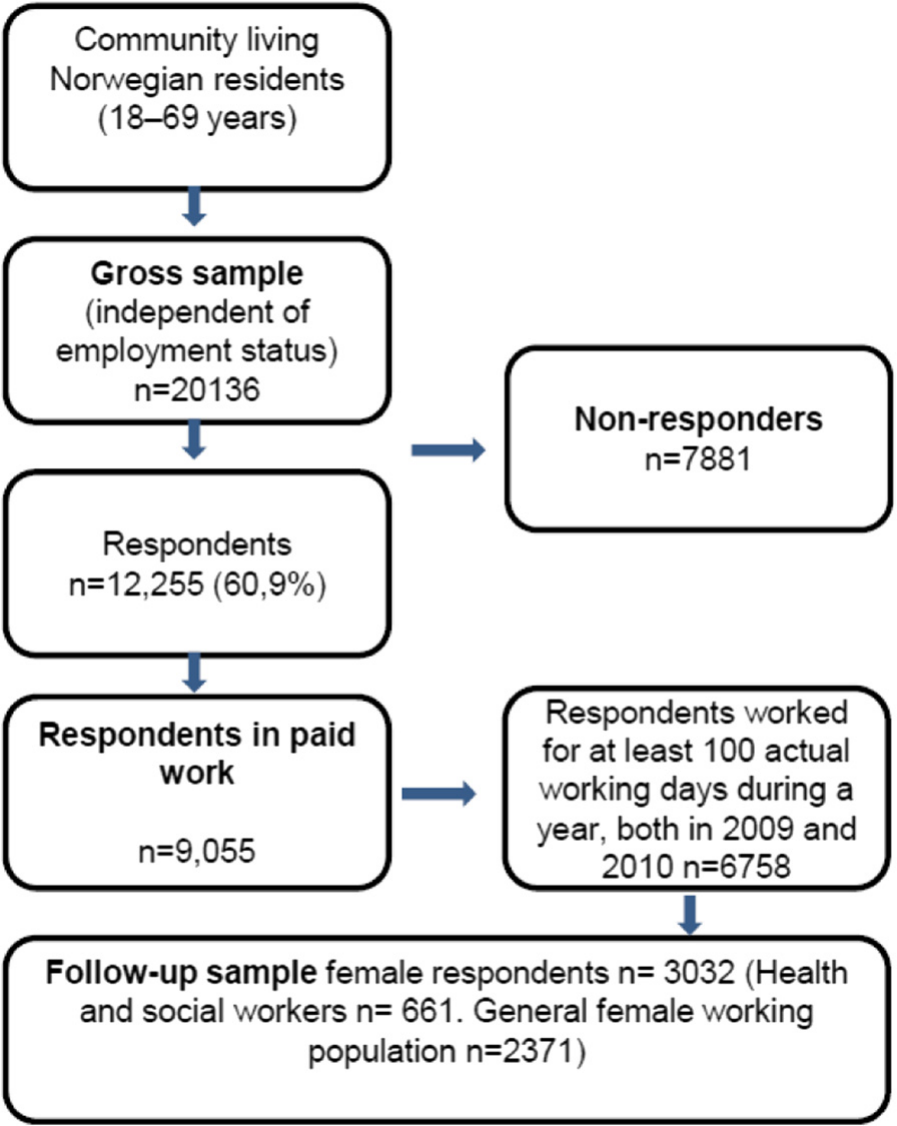
A flow chart of the selection process of respondents in the present study

Data were obtained from a nationwide study of the living conditions/work environment conducted by Statistics Norway. Eligible respondents were community-living Norwegian residents aged 18–69 years. In 2009, a gross sample of 20,136 was drawn randomly from this population and 12,255 (60.9 %) of these subjects were interviewed between 22 June, 2009 and 9 January, 2010 (Fig. 1). Data related to sick leave days were obtained from the Norwegian Labour and Welfare Administration’s sickness benefit register. This register includes all workers aged 16–69 years who live in Norway and who are registered with an active employee relationship.

### Study population

The follow-up sample in the present study (*n* = 3032) (Fig. 1) comprised female respondents who were in paid work for at least one hour during the reference week, or who were temporarily absent from such work, and who were registered with an occupation and an active employee relationship for at least 100 actual working days in each year (2009 and 2010). Using this sample, female health and social workers (*n* = 661) were compared with the general female working population (*n* = 2371).

## Sick leave

In Norway employees are entitled to use a personal declaration for sick leave of up to 3 days or a total of 8 days spread over four different occasions during a 12-month period, depending on their employer’s settlement with the Norwegian Labour and Welfare Organization. In addition the employee has the right to stay at home, if the worker’s child is sick. If the employee is sick beyond the personal declaration days, or if the severity of the illness requires it, then doctor-certified sick leave is required. Employees receive full compensation from the first day of sick leave. Because minor health problems such as influenza is covered by personal declaration days, we believe that doctor certified sick leave for 21 days or longer captures more serious sickness.

### Measurement

*LTSL* was defined as doctor-certified sick leave for a period of ≥21 actual working days during 2010, which was the year after the initial survey data were collected.

*Occupation* was based on an open questionnaire and coded by Statistics Norway as a professional title, in accordance with the International Standard Classification of Occupations (ISCO-88). First the professions included in the sample of female health and social workers was recoded into five occupational groups based on 4-digit code. Then the occupational groups in the female general working population were recoded into 26 occupational groups based on 2- digit code (Table 1).

**Table 1:** Distribution of responding women by occupational group

	Number	Percent
Health and social workers	661	21.5
Nurse	204	6.6
Physical therapist, Radiographer, Health worker with college	49	1.6
Social worker, social educator	87	2.8
Nursing and care assistants	254	8.3
Doctors-/dentists assistants, Pharmacy Technicans	67	2.2
Other occupations	2409	78.5
Legislators and senior officials in public administration and interest organisations	6	.2
Corporate managers of large and mediumsized enterprises	187	6.1
General managers of small enterprises	54	1.8
Physical, mathematical and engineering science professionals	83	2.7
Life science and health professionals	38	1.2
Teaching associate professionals	104	3.4
Public service administrative professionals	82	2.7
Other professionals	162	5.3
Engineering science associate professionals	69	2.2
Life science and health associate professionals	27	.9
Teaching associate professionals	298	9.7
Executive officers in administration, business services, social work and entertainment	414	13.5
Office clerks	226	7.4
Customer services clerks	26	.8
Personal and protective services workers	260	8.5
Models, salespersons and demonstrators	156	5.1
Agricultural workers	11	.4
Extraction and building trades workers	5	.2
Metal, machinery and related trades workers	12	.4
Precision, handicraft, printing and related trades workers	15	.5
Other craft and related trades workers	10	.3
Stationary-plant and related operators	7	.2
Machine operators and assemblers	30	1.0
Drivers and mobile-plant operators	7	.2
Services elementary occupations	80	2.6
Agricultural, fishery and related labourers	1	.0
Labourers in construction and manufacturing	2	.1
Unspecified or unidentified occupations (missing)	37	1.2
Total working population	3070	100.0

*Perceived psychosocial factors at work* included role conflict (three items, α = 0.64), low supportive leadership (three items, α = 0.70), job demands (two items, α = 0.70), job control (four items, α = 0.71),violence and threats of violence (three items), and bullying (two items), which corresponded to those used in previous studies [9, 14]. All of the factors were then treated as continuous variables (ranges 1–5), where high scores indicated unfavourable exposure, except for the dichotomous variables of violence and threats of violence and bullying. *Emotional demands* were measured by two items (α = 0.69): (i) “In your work, to what extent do you need to deal with strong feelings such as sorrow, anger, desperation and frustration from customers, clients or other people who are not employed at your workplace?”, where the response categories were “To a great extent”, “To some extent”, “Not really” and “Not at all”; and (ii) “In your work, to what extent do you need to conceal negative feelings such as anger, irritation and frustration from customers, clients or other people who are not employed at your workplace?”, where the response categories were “To a very great extent”, “To a great extent”, “To some extent”, “Not really” and “Not at all”. The five-point scale in question (ii) was recoded and converted to a four-point scale. The items were then collapsed into one variable for emotional demands (range 1–4) and treated as a continuous variable, where high scores indicated unfavourable exposure This item has been used in previous studies [9, 14]. *Perceived mechanical workload* was measured by eight items: neck flexion, hands above shoulders, hand/arm repetition, squatting/kneeling, standing, work with upper body bent forward, awkward lifting and heavy lifting. Scores were coded on a scale from 1 (not exposed or exposed very little in the working day) to 4 (exposed for three-quarters of the working day or more). All of the factors were treated as continuous variables. These items were described in greater detail in a previous study [11].

*Potential confounders* such as age and educational level were based on administrative registry data. Education was coded into five educational levels (years of education) and used as a continuous variable. Actual weekly working hours (working hours/week), including paid overtime and extra work done at home related to the main job, was collected from the survey data and were treated as a continuous measure. Previous LTSL defined as doctor-certified sick leave for a period of ≥21 actual working days during 2009, was collected from the national registry of social transfer payments.

### Statistical analysis

Correlations between variables were calculated with Pearson’s correlation coefficients. Univariate one-way analyses of variance were used to compare the mean scores for the self-reported work-related factors between women in health and social occupations and women in the general working population. Continuous variables were tested with *t*-tests and chi-squared tests were used for categorical variables. The associations between women in the health and social occupations and women in the general working population with LTSL were calculated as the odds ratio (OR) and 95 % confidence interval (95 % CI). Multiple regression analyses were conducted in the following sequence. First, in the initial model, we adjusted for age, education, previous LTSL and working hours/week. Second, we added each psychosocial factor one at a time. Third, we adjusted for all of the psychosocial factors simultaneously. The same procedure was applied to the work-related mechanical factors. Finally, we added all of the factors simultaneously. The impact (%) of each separate factor or set of factors on the occupational differences was estimated as follows: (OR_adjusted_ – OR _initital_)/(OR _initital_ – 1) × 100 (percentage of change in OR in the initial model). Statistical analyses were conducted using SPSS Statistics for Windows version 21.0 (IBM Corporation, Armonk, NY, USA).

The survey was carried out by Statistics Norway according to statutory rules. Statistics Norway has appointed its own privacy ombudsman, who is approved by the Norwegian Data Inspectorate. All subjects gave their informed consent prior to inclusion in the study [15].

## Results

In total, 24.1 % (159/661) of the women in health and social occupations and 17.9 % (425/2371) of those in the general working population were classified with LTSL (chi-squared test, *p* < 0.001). There were no significant differences attributable to age or educational level between the two groups, but women in health and social occupations reported a lower mean level of working hours/week (33.0 versus 37.1 h/week) (*p* < 0.001) (Table 1). Women in health and social occupations reported higher levels for six out of eight psychosocial factors, where the largest differences were observed in terms of emotional demands, violence and threats of violence, role conflict and job control. No significant differences were found for job demands and bullying. For mechanical factors, women in health and social occupations reported higher levels in six out of eight factors, where the largest differences were found for standing, heavy lifting, awkward lifting and upper body bent forward. For hand/arm repetition, a lower mean level was reported in women working in health and social occupations compared with other working women, but there was no significant difference between the groups for hands above the shoulder. Correlations between the variables were negligible to moderate. The correlation coefficients between the psychosocial factors ranged from *r* = 0.02 to *r* = 0.32. The correlation coefficients between the mechanical factors ranged from *r* = 0.06 to *r* = 0.43. Finally, correlation coefficients between psychosocial factors and all mechanical factors ranged from *r* = 0.01 to *r* = 0.14 (Table 2).

**Table 2:** Descriptions of sick leave and explanatory variables for women in health and social occupations (*n* = 661) and women in the general working population (*n* = 2371)

		Health and social occupations		General working population		
	Range	Mean	SD	Mean	SD	*p*-value^a^
Outcome variable						
Long-term sick leave (LTSL)	0–1	0.24	0.43	0.18	0.38	0.001
Age	18–69	44.51	10.82	43.65	11.33	0.081
Working hours/week	0–90	33.05	8.46	37.06	7.53	0.001
Educational level	1–5	3.31	0.93	3.24	1.2	0.151
Previous LTSL	0–1	0.23	0.42	0.16	0.36	0.001
Psychosocial factors						
Violence and threats of violence	0–1	0.25	0.43	0.05	0.22	0.001
Emotional demands	1–4	3.26	0.87	2.41	0.94	0.001
Role conflict	1–5	2.23	0.84	2.07	0.83	0.001
Supportive leadership	1–5	2.07	0.96	1.92	0.88	0.001
Job demand	1–5	3.74	0.96	3.75	0.9	0.981
Job control	1–5	2.85	0.7	2.59	0.8	0.001
Bullying	0–1	0.29	0.17	0.28	0.16	0.917
Possibilities of development	1–5	1.88	0.58	1.78	0.6	0.001
Mechanical factors						
Neck flexion	1–4	1.51	0.85	1.4	0.86	0.001
Hand/arm repetition	1–4	1.69	1.06	2.17	1.32	0.001
Hands above shoulder	1–4	1.15	0.52	1.19	0.59	0.142
Squatting/kneeling	1–4	1.31	0.63	1.19	0.58	0.001
Standing	1–4	2.79	1.22	2.1	1.28	0.001
Upper body bent forward	1–4	1.37	0.72	1.14	0.52	0.001
Awkward lifting	1–4	1.35	0.67	1.14	0.5	0.001
Heavy lifting	1–4	1.55	0.85	1.13	0.47	0.001

For categorical variables (range 0–1), the mean score equals the proportion of respondents registered with a value of 1 (i.e., the percentage of respondents who were exposed)

^a^Continuous variables were tested with *t*-tests and chi-square tests were used for categorical variables

In the initial model (adjusted for age, educational level, previous LTSL and working hours/week), women in health and social occupations had a significantly higher risk of LTSL compared with women in the general working population (OR = 1.42; 95 % CI = 1.13–1.79, *p* = 0.003) (Table 3). Adjusting for psychosocial factors reduced the OR by 57 %. The most important factors were violence and threats of violence (36 %) and emotional demands (28 %). Adjusting for all mechanical factors reduced the OR by 24 %, where the most important factor was awkward lifting (21 %), although upper body bent forward, standing and heavy lifting were also significant. When all of the variables were entered simultaneously, psychosocial and mechanical factors explained 70 % of the increased risk for LTSL in women in health and social occupations compared with women in the general working population.

**Table 3:** Multiple logistic regression for long-term sick leave (LTSL) regressed on women and the effects of adjusting for mechanical and psychosocial working conditions (OR = odds ratio; 95 % CI = 95 % confidence interval)

	LTSL	
Initial model^a^	OR (95 % CI)^a^	% Change^c^
General working population (n = 2371 (17.9))^b^	1.0	
Health and social sector (n = 661 (24.1))^b^	1.42 (1.13–1.79)^d^	
Psychosocial factors		
Violence and threats of violence	1.27 (0.99–1.61)	−0.36
Emotional demands	1.32 (1.03–1.68)	−0.25
Role conflict	1.39 (1.10–1.75)	−0.08
Supportive leadership	1.40 (1.11–1.76)	−0.05
Job demand	1.43 (1.14–1.79)	0.01
Job control	1.40 (1.11–1.76)	−0.06
Bullying	1.42 (1.12–1.79)	−0.002
Possibilities of development	1.41 (1.12–1.74)	−0.03
All psychosocial factors	1.18 (0.92–1.52)	−0.57
Mechanical factors		
Neck flexion	1.42 (1.13–1.42)	0
Hand/arm repetition	1.47 (1.16–1.85)	0.12
Hands above shoulder	1.45 (1.16–1.83)	0.07
Squatting/kneeling	1.41 (1.22–1.73)	−0.02
Standing	1.36 (1.08–1.71)	−0.14
Upper body bent forward	1.35 (1.07–1.70)	−0.16
Awkward lifting	1.33 (1.06–1.68)	−0.21
Heavy lifting	1.37 (1.08–1.73)	−0.12
All mechanical factors	1.32 (1.03–1.69)	−0.24
All variables included	1.13 (0.87–1.48)	−0.7

^a^Adjusted for age, LTSL in 2009, education and working hours/week

^b^Number of respondents (cases of LTSL, %)

^c^Percentage change in OR after comparing the initial OR with the further adjusted OR (i.e., the initial OR adjusted for work-related factors)

^d^ *p* = 0.003

## Discussion

This study examined the roles of work-related psychosocial and mechanical factors in explaining the difference in doctor-certified LTSL between women in health and social occupations compared with women in the general working population. In the initial model, after adjusting for age, education, previous LTSL and working hours/week, we detected a 40 % higher risk for LTSL among women in health and social occupations. After adjustment, psychosocial and mechanical factors explained about 70 % of this increased risk for LTSL in female health and social workers. Psychosocial factors were most important, particularly violence and threats of violence, and emotional demands. In addition, mechanical factors such as awkward lifting, as well as working with the upper body bent forward were important contributors to the increased risk. All of these work environment factors are prevalent in occupations that involve handling patients or clients in health and social care, and our results indicate that these factors are of great importance in explaining the higher risk of LTSL among women in this particular sector.

A novel finding of our study was that both psychosocial and mechanical factors appeared to explain a substantial part of the higher risk of LTSL in health and social occupations compared with women in the general working population. Previous studies of the general working population have reported that psychosocial factors explained nearly 30 % of the increased risk for LTSL among women compared with that among men, whereas mechanical factors were negligible [16, 17]. The results of our study indicate that comparisons of more specific populations can provide additional knowledge about particular risk factors, which may account for the high level of sick leave in some female-dominated occupations.

Our finding that psychosocial factors were more important than mechanical factors in explaining the higher risk of sick leave among female health and social workers supports previous studies, which reported that psychosocial factors were important risk factors for LTSL in these occupations [4, 7, 8]. It is possible that psychosocial factors, such as violence and threats of violence and emotional demands, are characteristics of this sector, whereas mechanical exposure is more widespread and common in various occupational groups. However, due to the relatively low sample size in the health and social occupations, it was not possible to analyse the different occupational groups separately. Heavy lifting, awkward lifting and working with the upper body bent forward may be prevalent in some health and social occupations, but exposure to these factors is less prevalent in some occupations such as social workers, doctor/dentist assistants and pharmacy technicians. Thus, we cannot exclude the possibility that mechanical factors are more important for LTSL in some health and social occupations than others, which requires further investigation.

In the present study, we found that factors in the work environment helped to explain about two-thirds of the higher risk for sick leave among women in health and social occupations. Thus, one-third of the increased risk could be explained by other factors. However, we cannot rule out the possibility that unmeasured work-related factors or factors outside the workplace may have contributed further to explain the difference. The gender composition of the workplace has been discussed as a potential explanation for the increased risk of LTSL in this sector, and some studies have indicated that sick leave is higher in occupations or workplaces dominated by one gender, particularly in strongly female-dominated occupations [18, 19]. Gender-balanced occupations with 40–60 % women have been shown to have the lowest level of sickness absence [20]. In agreement with this reasoning, a 2010 report by the Ministry of Health and Care Services on sickness absence in the health and social care sector in Norway suggested that the increased risk for sick leave in this sector may be explained by the fact that this sector is female dominated and that women in general have a high risk for sick leave [2]. We cannot exclude the possibility that the gender composition contributed to some of the higher risk for LTSL among female health and social workers, but we performed separate analyses for women and our results suggested that work environment factors were of great importance in explaining the higher risk of LTSL among women in this sector.

The main strength of this study was the use of a large nationwide survey based on random sampling, where we measured a comprehensive set of work-related psychosocial and mechanical exposure factors, which were prospectively linked to registered sickness absence data, with practically no loss to follow-up. The use of different sources for measures excludes the potential for common method bias [21]. Nevertheless, reporting bias cannot be excluded because of the self-reported assessment of the explanatory variables and other covariates.

The study had a fairly high response rate of 61 %. After evaluating potential systematic differences between responders and non-responders, Statistics Norway found no differences across the benchmarks of age, sex and region [15]. On the other hand, we do not know whether people with poor health, or elevated risk for sick leave, were less likely to respond at baseline, which may have led to biased and attenuated estimates and thus threatened the internal validity. However, studies have shown that some differences in participation in questionnaire surveys related to socio-demographic variables and health status do not produce biased risk estimates [22, 23]. The outcome variable of LTSL was registered during the year after we measured exposure to work-related psychosocial factors using a survey questionnaire. A longer follow-up time could have the advantage of providing more sufficient time of exposure to create effects on the outcome variable. However a longer follow-up time could be considered a limitation as well, due to the fact that during a longer time period between exposures and effect, the levels of exposure might have changed for some participants, which may lead to an underestimation of effect sizes [4]. Thus, the ideal time-lag for longitudinal job stress research, has remained a long standing methodological issue, and definitive insights remain indefinable to-date [24] In this present study the cut-off chosen to define LTSL (≥21 days) during a calendar year was considered a reasonable proxy for LTSL and one that allowed us to compare our findings with those of other studies of psychosocial predictors of LTSL. In a 2004-review, work -environment factors were considered more important for long-term than short-term sick leave [25]. However, in general it is challenging to make comparisons between studies due to the use of different definitions of sick leave.

## Conclusion

In conclusion, exposure to both psychosocial and mechanical risk factors contributed significantly to explaining the difference in doctor-certified LTSL between women in health and social occupations and women in the general working population. The main factors that contributed to the higher risk of LTSL were violence and threats of violence, and emotional demands. In addition, mechanical factors made important contributions to the higher risk for LTSL among women in this specific sector. The results from this study highlight the importance of the work environment in explaining the increased risk for sick leave among women in health and social occupations. Interventions aimed at reducing LTSL in these occupations may benefit from focusing on specific psychosocial and mechanical risk factors in the work environment.

## Abbreviation

LTSL: long-term sick leave
